# A New and Robust Method of Tethering IgG Surrogate Antigens on Lipid Bilayer Membranes to Facilitate the TIRFM Based Live Cell and Single Molecule Imaging Experiments

**DOI:** 10.1371/journal.pone.0063735

**Published:** 2013-05-22

**Authors:** Shaosen Zhang, Liling Xu, Xingwang Zhao, Xin Chen, Yilin Fan, Zhengpeng Wan, Yinsheng Xu, Wanli Liu

**Affiliations:** 1 MOE Key Laboratory of Protein Science, School of Life Sciences, Tsinghua University, Beijing, China; 2 Collaborative Innovation Center for Infectious Diseases, Beijing, China; 3 School of Medicine, Tsinghua University, Beijing, China; National Cancer Institute at Frederick, United States of America

## Abstract

Our understanding of cell-cell interactions has been significantly improved in the past years with the help of Total Internal Reflection Fluorescence Microscope (TIRFM) in combination with an antigen presenting system supported by planar lipid bilayer (PLB) membranes, which are used to mimic the extensive receptor and ligand interactions within cell-cell contact interface. In TIRFM experiments, it is a challenge to uniformly present ligand molecules in monomeric format on the surface of PLB membranes. Here, we introduce a new and robust method of tethering IgG surrogate antigen ligands on the surface of Ni^2+^-containing PLB membranes. In this method, we use a modified D domain from staphylococcal protein A molecule that is fused with an N-terminus polyhistidine tag (H12-D-domain) to tether IgG surrogate antigens on Ni^2+^-containing PLB membranes. We systematically assessed the specificity and capability of H12-D-domain construct to capture IgG molecules from different species through live cell and single molecule TIRFM imaging. We find that these IgG surrogate antigens tethered by H12-D-domain show better lateral mobility and are more uniformly distributed on PLB membranes than the ones tethered by streptavidin. Neither IgM molecules, nor Fab or F(ab’)_2_ fragments of IgG molecules can be tethered on PLB membranes by H12-D-domain construct. These tethered IgG surrogate antigens strongly induce the formation and accumulation of signaling active antigen receptor microclusters within the immunological synapse in B or T lymphocyte cells. Thus our method provides a new and robust method to tether IgG surrogate antigens or other molecules fused with IgG Fc portion on PLB membranes for TIRFM based molecule imaging experiments.

## Introduction

Cell-cell contact based information exchanges play key roles in maintaining the function of various types of organismal systems, for instance, the neurological system and the immunological system. Within cell-cell contact interfaces, extensive receptor and ligand interactions are established. It is not completely clear how these interactions mediate the formation and stability of cell to cell adhesion focal planes. Neither clear is how these interactions initiate and modulate cross membrane signal transduction. These types of questions have been attracting the research interests of many laboratories. To visualize these receptor and ligand interactions within such cell-cell contact interface, a variety of advanced fluorescence microscope techniques have been applied to the related studies [1]–[4]. Among them, live cell and single molecule imaging approaches through the Total Internal Reflection Fluorescence Microscope (TIRFM) made unique and important contributions. TIRFM based imaging techniques can visualize molecular events on or proximal to plasma membrane of a cell with a superior signal-to-noise ratio because the depth of optical section in TIRFM is very thin, just 100 nm [4]. With the help of these state-of-the-art live cell imaging techniques including TIRFM, our understanding of cell-cell interactions has been significantly advanced in the past years. For instance, the pioneer studies on immune cell-cell interactions demonstrated the hierarchical complexity of immunological synapse supramolecular activation clusters (SMAC) including central SMAC (cSMAC), peripheral SMAC (pSMAC) and distal SMAC (dSMAC) [5]–[8]. Within each hierarchy of these SMAC structures, distinct immune receptor and ligand interactions show up and play different roles in maintaining an appropriate extent of immune cell activations [9]–[11].

In TIRFM imaging experiments, an antigen presentation system supported by fluid planar lipid bilayer (PLB) membranes has been widely used to mimic the cell-cell contact interface as recently reviewed by Dustin and colleagues [12]. PLB membranes have a long history to serve biological studies. In 1980s, McConnell and colleagues tethered phospholipid hapten or IgE molecule to PLB membranes, and used such membrane to elegantly capture the aggregation of laterally mobile IgE molecules into microaggregates upon contact of mast cells expression Fcε receptors to these PLB membranes [13]–[15]. However it is probably until the combination of PLB membrane based antigen presentation system and the advanced TIRFM imaging technique that the value of PLB membrane based antigen presentation system has been best explored [16]. For example, in the studies of T cell immunological synapse, TIRFM imaging experiments on PLB membranes tethering adhesion molecule and TCR antigens revealed the formation of LFA-1 and TCR microclusters, and the concomitant involvement of negative regulators including CTLA-1 and PD-1 microclusters [16]. More importantly, it was discovered that the TCR-p-MHC and CD28-CD80 binding partners mainly localized in the cSMAC area, while LFA-1 and its ligand ICAM-1 mainly localized in the pSMAC area [17]. Using a similar imaging system, significant progress was also recently achieved in the studies of B cell immunological synapse and B cell antigen microclusters by our studies and those of others [8], [18]–[26].

For these TIRFM imaging experiments, in many cases, the investigators need to tether IgG molecules as surrogate antigens on the surface of PLB membranes to mimic the function of physiological antigens. For example, in T cell studies, anti-CD3 or anti-CD28 antibodies are widely used as surrogate antigens to activate the T cells; similarly, in B cell studies, anti-IgM, anti-IgD and anti-IgG antibodies are widely used as surrogate antigens to activate the B cells. The current solution is to have these antibodies biotinylated and then have them tethered on PLB membranes containing biotinylated lipid using streptavidin as a linker molecule. Although this solution is widely used by others and us, it is not very convenient as the biotin-conjugated IgG molecule instead of intact IgG needs to be used. When biotinylating the IgG molecule, it is usually difficult to control where on the surface of IgG molecules to conjugate the biotin molecules, thus the biotin molecules can be covalently conjugated to either the variable region or the constant region of the IgG molecules. If the biotin is conjugated to the light chain or the variable region of IgG heavy chain, it is highly possible that the biotinylated IgG molecules are tethered on biotin-containing PLB membranes in an “upside down” manner. In this case, the “upside down” format of IgG surrogate antigens on PLB membranes is not easily accessible to the receptors.

In this report, we introduce an alternative method to tether IgG molecules or other recombinant proteins fused with an IgG Fc portion on the surface of PLB membranes. In our method, instead of using PLB membranes containing biotinylated lipids, we used Ni^2+^-containing PLB membrane which is also commonly used in TIRFM imaging experiments [24], [25], [27]. In this alternative method, we chemically synthesized a modified version of the D domain of staphylococcal protein A molecule fused with an N-terminus His12 tag (H12-D-domain) to tether IgG molecules of interest on Ni^2+^-containing PLB membranes. The D domain of staphylococcal protein A molecule has been well studied to show the capability to bind the constant region of IgG molecule in a 1 to 1 ratio [28]. And protein A molecules have been widely used to purify IgG molecules from sera. Here, using B or T lymphocyte cells as examples, we systematically assessed the specificity and capability of H12-D-domain construct to capture IgG molecules from different species. Through live cell and single molecule TIRFM imaging, we found that these IgG surrogate antigens show good lateral mobility and are uniformly distributed on PLB membranes. Neither IgM molecules, nor Fab or F(ab’)_2_ fragments of IgG molecules can be tethered on PLB membranes by H12-D-domain construct. More importantly, we determined that these IgG surrogate antigens well maintained their stimuli characteristics. Upon encountering these IgG surrogate antigen-containing PLB membranes, B lymphocyte cells showed the formation and accumulation of signaling active antigen receptor microclusters within the immunological synapse, and efficiently up-regulated the cell surface activation maker CD69. In summary, our reported method using H12-D-domain provides a new way to tether the IgG surrogate antigens or other recombinant proteins fused with IgG Fc portion on PLB membranes for TIRFM based molecule imaging experiments.

## Materials and Methods

### Cells, Antibodies and Peptide

Human Ramos B cells and mouse A20 B cells were cultured in RPMI 1640 medium containing 10% FBS, penicillin and streptomycin antibiotics (Invitrogen) as described [25]. Mouse EL4T cells were cultured in DMEM medium containing 10% FBS (Invitrogen). Chicken DT40 B cells were cultured in RPMI 1640 medium containing 10% FBS, 1% chicken serum, 50 µM 2-mercaptoethanol, penicillin and streptomycin antibiotics (Invitrogen). Human Ramos B cells, mouse A20 B cells and chicken DT40 B cells were gifts for laboratory scientific studies from Dr. Susan K. Pierce (NIAID-NH). Mouse EL4T cells were gifts from Dr. Hai Qi (Tsinghua University, Beijing, China). All these four cell lines were originally purchased from ATCC of USA. Mice primary B cells were isolated from spleens of BALB/c mice as reported [23]–[25].

Most of the antibodies used in this paper were purchased from Jackson ImmunoResearch, Rockland Immunochemicals or SouthernBiotech including Alexa Fluor 647-conjugated Goat IgG anti-human IgM, FITC-conjugated Mouse IgG anti-Chicken IgM, Alexa Fluor 647-conjugated Donkey IgG anti-mouse IgM, FITC-conjugated Rabbit IgG anti-mouse IgG, FITC-conjugated Rat anti-mouse CD3 molecular complex, Alexa Flour 647-conjugated Goat IgG anti-human IgM, Alexa Fluor 568-conjugated F(ab’)_2_ fragment of Goat IgG, Alexa Fluor 488-conjugated Fab fragment of Goat IgG, Mouse IgM anti-Chicken IgM, Alexa Fluor 405-conjugated Rabbit IgG anti-mouse IgG. Alexa Fluor 568-conjugated Streptavidin, Alexa Fluor 647-conjugated F(ab’)_2_ fragment of Goat IgG anti-Rabbit IgG were purchased from Invitrogen. FITC-conjugated Hamster IgG anti-Mouse CD69 (H1-2F3) was purchased from BD. We conjugated both biotin and Alexa 568 to goat whole IgG anti-human IgM antibodies following our published protocol [23]–[25]. H12-D-domain polypeptides were chemically synthesized by ShangHai Leon Chemical Ltd (ShangHai, China) and were HPLC purified and verified by mass spectroscopy with >90% purity.

### Preparation of Fluid PLB Membranes Tethering H12-D-domain

Planar lipid bilayer membranes were prepared following a well-established method [24], [29], [30]. For Ni^2+^-containing lipids, 1,2-dioleoyl-sn-glycero-3-phosphocholine (DOPC) and 1,2-dioleoyl-sn-glycero-3-[N(5-amino-1-carboxypentyl) iminodiacetic acid]-succinyl (nickel salt; DOGS–Ni-NTA; Avanti Polar Lipids, Inc.) were mixed at a 9∶1 ratio of DOPC:DOGS–Ni-NTA. For biotin-containing lipids, lipids were made by mixing 99% 1,2-dioleoyl-sn-glycero-3-phosphocholine lipid and 1% 1,2-Dioleoyl-sn-Glycero-3-phosphoethanolamine-cap-biotin (Avanti Polar Lipids). The mixed lipids were sonicated to form unilamellar vesicles. After overnight ultra-centrifuge and filtration, small unilamellar vesicles (SUV) were acquired and used as stock SUV resolution following our published protocol [24]. The SUV was added at a final concentration of 0.1 mM to the Lab-Tek chambers (Nalge Nunc). The original cover slide on the Lab-Tek chamber was replaced by an acidic buffer-washed coverslip. After an incubation time of 30 min at room temperature (RT), the unbound SUV lipids were thoroughly washed away. H12-D-domain (0 or 50 nM) was attached to the Ni-NTA-containing PLB membranes for 20 min at 37°C. After thorough washing and blocking with 5% BSA in PBS, the PLB membranes tethering H12-D-domain constructs were ready for later use.

### IgG Surrogate Antigens Bind to the PLB Membranes through H12-D-domain

To test the binding specificity of IgG molecules on PLB membranes mediated through H12-D-domain constructs, 100 nM Alexa Fluor 647 Goat IgG anti human IgM, or 100 nM Alexa Fluor 647-conjugated Donkey IgG anti-mouse IgM, or 100 nM FITC-conjugated Rabbit IgG anti-mouse IgG were loaded to PLB membranes tethering 50 nM or 0 nM (a negative control) H12-D-domain construct for 20 min at RT and washed thoroughly. The binding of these fluorophore conjugated IgG surrogate molecules were detected by TIRFM imaging subsequently. To further detect the binding specificity of IgG molecules on PLB membranes tethering H12-D-domain construct, 100 nM Alexa Fluor 568-conjugated F(ab’)_2_ fragment of Goat IgG, or 100 nM Alexa Flour 488 conjugated-Fab fragment of Goat IgG, or 100 nM biotin-conjugated mouse IgM molecules were loaded to PLB membranes tethering 50 nM H12-D-domain construct for 20 min at RT and washed clearly; in the case of IgM group, 50 nM Alexa568 streptavidin was further added for another 30 min at 37°C and washed thoroughly. The binding of these molecules was detected by TIRFM imaging subsequently. To test the binding capability of IgG molecules on PLB membranes mediated through H12-D-domain constructs, Alexa Fluor 647-conjugated goat IgG anti-Human IgM (0 nM, 50 nM or 100 nM), or FITC-conjugated Mouse IgG anti-Chicken IgM (0 nM, 25 nM, 50 nM, 100 nM) were incubated with the PLB membranes tethering H12-D-domain for 20 min at RT and washed thoroughly. The binding was detected by TIRFM imaging.

### Molecule Imaging by Total Internal Reflection Fluorescence Microscope (TIRFM)

Molecule images were acquired by an Olympus IX-81 microscope supported by a TIRF port, ANDOR iXon+ DU-897D electron-multiplying EMCCD camera, Olympus 100x 1.45 NA objective TIRF lens, a 488 nm, a 561 nm and a 633 nm laser (Sapphire lasers, Coherent). The exposure time was 100 ms unless specially indicated. Acquisition was controlled by Metamorph software (MDS Analytical Technologies). Images were analyzed by Image Pro Plus (Media Cybernetics), Image J (NIH, U.S.), or Matlab (Mathworks) software following our published protocol [23]–[26].

### Accumulation of BCR, TCR and Antigen Microcluster into the Immunological Synapse

To check the biological activity of these IgG surrogate antigens tethered on PLB membranes, live B cells or T cells were placed on these IgG molecules-containing PLB membranes, and the responses of these lymphocytes cells were examined by TIRFM as reported earlier [23]–[26]. Briefly, the formation and accumulation of BCR, TCR and IgG surrogate antigen microclusters into the immunological synapse of the cells’ contact with the IgG molecule-containing PLB membranes were examined. The mathematical analyses and quantification of the accumulation of the TIRFM images were processed following our published protocols [23]–[26].

### Intracellular Immunofluorescence Staining and Molecule Imaging of pSyk within the Immunological Synapse

Recruitment of pSyk into the immunological synapse of human Ramos B cells upon encountering IgG surrogate antigens tethered on PLB membranes was imaged by TIRFM following our published protocol with modifications [23], [24]. Briefly, Ramos cells were fixed with 4% paraformaldehyde 10 min after incubation with the PLB membranes containing IgG surrogate antigens. We will suggest including a step to have the H12-D-domain constructs on PLB membranes blocked with high concentration of purified goat IgG molecules (or IgG molecules from other species depending on the usage of secondary antibodies). The fixed B cells were permeabilized with 0.1% Triton X-100 and pretreated with blocking reagent including 1% FBS and 1% goat sera. Subsequently, Ramos cells were stained with Phospho-Zap-70 (Tyr319)/Syk (Tyr352) antibody (Cell Signaling Technology), followed by secondary antibody Alexa Fluor 647-conjugated F(ab’)_2_ goat antibodies specific for rabbit IgG (Invitrogen) staining as previously described [23], [24]. Images were analyzed by Image J (NIH, U.S.) and Matlab (Mathworks) software following our published protocols [23]–[26].

### Flow Cytometry Analysis of B Cell Activation

Primary B cells were isolated from spleens of BALB/c mice as reported [23]–[25]. Cells were then counted and seeded at 1×10^6^ cells/ml in RPMI 1640 containing 10% FBS, then loaded into PLB membranes containing IgG surrogate antigen molecules. After culturing for 24 hours at 37°C with 5% CO_2_, cells were harvested. Before harvesting the cells, we will recommend including a step to have the H12-D-domain constructs on PLB membranes blocked with high concentration of purified goat IgG molecules. The harvested B cells were stained with FITC-conjugated Hamster anti-Mouse CD69 for 15 min on ice, then washed twice and accessed by flow cytometry.

### Single Molecule Imaging by TIRFM and the Analyses

To check the mobility of IgG surrogate molecules tethered on PLB membranes, we did single molecule imaging experiment by TIRFM following our published protocol [23]–[25]. Briefly, fluorophore conjugated IgG molecules tethered on PLB membranes were imaged using TIRFM with a 488 nm laser at an output power of 5 mW at the objective lens in epi-fluorescence mode. A sub-region of roughly 100×100 pixels was used to achieve an exposure time of 20 ms. Individual IgG surrogate antigen molecules was captured on 300 frames in a time course of 10 s in streamline acquisition mode. Single-molecule tracking was made using Matlab code based on available positional fitting and tracking algorithms [31], [32]. MSD and short-range diffusion coefficients for each IgG molecule trajectory were calculated from positional coordinates, as previously reported [32], [33], and plotted as CPD graphs. In this study, ten thousands of single IgG molecules trajectories were acquired and shown.

### Ethics Statement

The animal work and the collection of spleen samples were following an animal protocol that was reviewed and approved by the Institutional Animal Care and Use Committee (IACUC) of Tsinghua University. The Assurance Identification Number is 12-LWL1 and was issued by Dr. Zai Chang, the vice chair of IACUC of Tsinghua University, Beijing, China.

## Results

### The Design of a Modified Version of H12-D-domain to Bind to Ni^2+^-containing PLB Membranes

**Figure 1:**
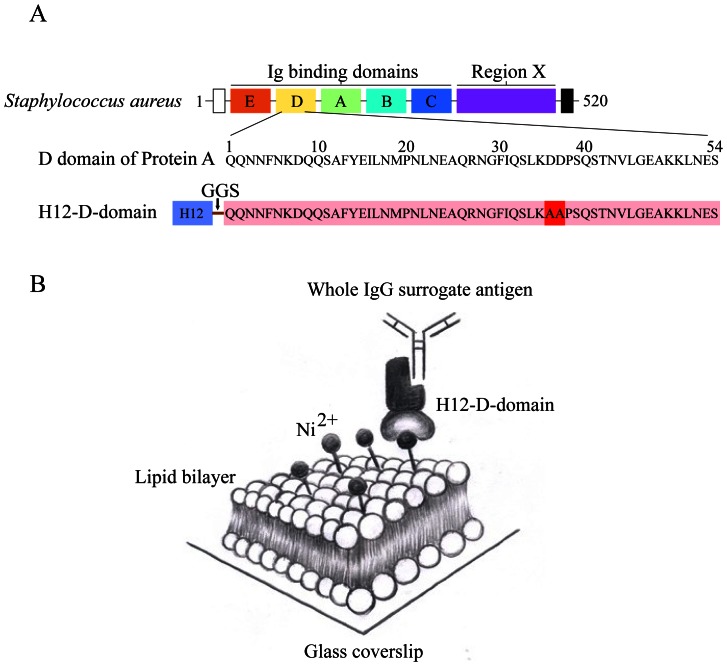
Schematic presentation of the amino acid sequences of H12-D-domain and its linker function of tethering IgG surrogate antigens on Ni^2+^-containing PLB membranes. (A) All the domains within the protein A molecule from *Staphylococcus aureus* [36]. One protein A molecule has five 56 to 61 aa residue Ig binding domains (domain A, B, C, D and E respectively). The amino acid sequences of D domain of Protein A are given. Also given are the amino acid sequences of the modified version of H12-D-domain used in the new method. H12 stands for HHHHHHHHHHHH polyhistidine tag. To eliminate the Fab VH-3 region binding capability of D domain, we chose to mutant the DD (aa36–37) to AA (aa36–37) in the H12-D-domain construct. (B) A cartoon to present the function of H12-D-domain construct to link the intact whole IgG surrogate antigen molecule on the surface of Ni^2+^-containing PLB membranes on supported glass coverslips.

The core purpose of this paper is to introduce a molecule that specifically binds the intact whole IgG molecule with high affinity in a 1 to 1 ratio. We draw our attention to the various types of gram-positive bacteria IgG-binding receptor proteins showing the well-established binding feature to mammalian immunoglobulins (Igs) [34]–[37]. One type of such bacteria IgG-binding receptor protein is the Protein A protein from *Staphylococcus aureus* [36]. One protein A molecule has four or five 56 to 61 aa residue Ig binding domains (domain A, B, C, D and E respectively) (Fig. 1A). Each such Ig binding domain folds into triple helical bundles (H1, H2 and H3) linked with short amino acid sequences [28]. Structural biological studies demonstrated that through these Ig binding domains, protein A strongly binds to the Fc portion of IgG molecules, but also binds to the Fab VH3 region of both IgG and IgM molecules [38]. Due to these facts, we cannot use whole protein A to satisfy our needs. We thus made some modifications to protein A molecules. These five Ig binding domains of staphylococcal protein A molecule showed high homology rate. Through the atomic level interaction analyses, the recent studies of Olaf and colleagues [38] showed that for each of these five Ig binding domains, the Glutamine (Q) 9 and 10 in H1 helical bundle are critical for the binding to Fc portion of IgG molecules, while the Aspartate (D) 36 and 37 in the linker region between H2 and H3 helical bundle are critical for the binding to the Fab VH3 region of both IgM and IgG molecules (Fig. 1A). Indeed a mutant construct of the D domain of staphylococcal protein A molecule, with the QQ (aa9–10) and DD (aa36–37) mutated to KK (aa9–10) and AA (aa36–37), completely lost the binding capability to the Fc portion of human IgG and the Fab VH3 region of both human IgM and IgG molecules [38], [39].

Based on these elegant early studies, we came up with a modified version of the D domain of staphylococcal protein A molecule (Fig. 1A). Since we need to maintain the IgG Fc portion binding capability and need to eliminate the Fab VH3 region binding capability, we choose to mutate the DD (aa36–37) to AA (aa36–37) in the modified D domain sequences (Fig. 1A). In addition we also need to provide this modified D domain construct the capability to specifically bind to Ni^2+^-containing PLB membranes, we fused a 12 aa polyhistidine tag at the N-terminus of the modified D domain, showing the following amino acid sequence as shown in Figure 1A, HHHHHHHHHHHH-GGS-QQNNFNKDQQSAFYEILNMPNLNEAQRNGFIQSLKAAPSQSTNVLGEAKKLNES (termed as H12-D-domain construct in this report). Instead of purifying the modified H12-D-domain construct from *E. coli* cells, we choose to chemically synthesize the H12-D-domain construct because of its relative small size with only 69 aa residues (Fig. 1A). The vendor we used easily synthesized the H12-D-domain construct with over 90% purify per mass-spec and HPLC quality control. We used these synthesized H12-D-domain molecules for all the imaging experiments performed in this report.

### H12-D-domain Construct Efficiently Tethers the IgG Surrogate Antigens on PLB Membranes in a Dose Dependent Manner

**Figure 2:**
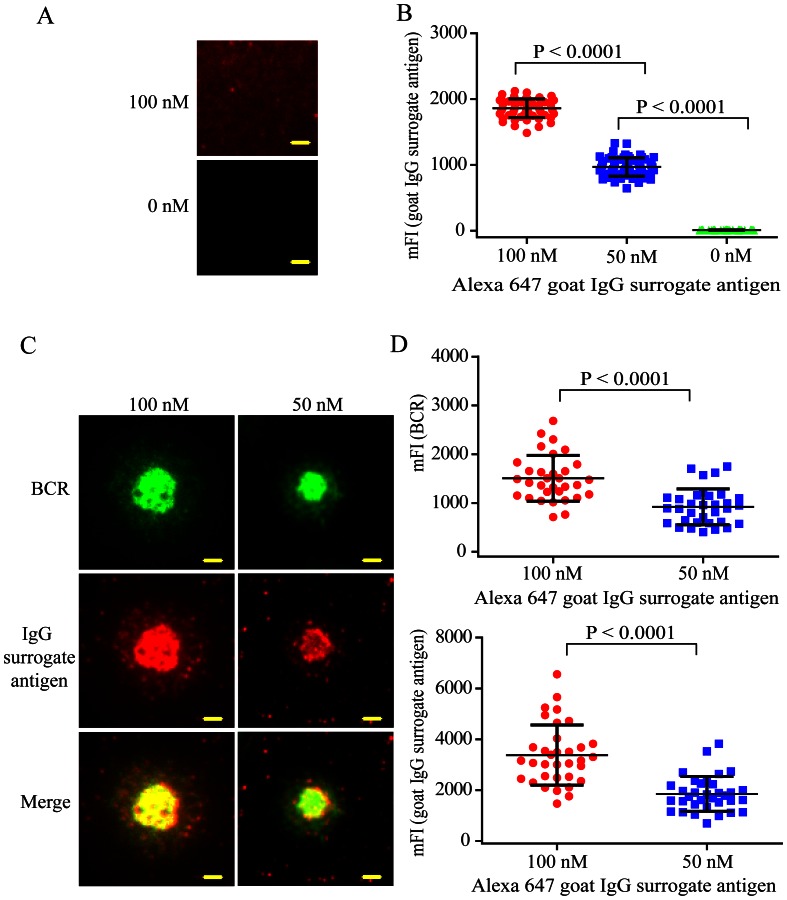
H12-D-domain construct efficiently tethers the IgG surrogate antigens on PLB membranes and these tethered IgG surrogate antigens induce the formation of BCR and surrogate antigen microclusters. (A) Shown are representative TIRFM images of Alexa 647-conjugated goat IgG anti human IgM surrogate antigens tethered on the surface of PLB membranes through H12-D-domain. The Alexa 647-conjugated goat IgG anti human IgM surrogate molecules were pre-incubated (100 or 0 nM) with the PLB membranes containing H12-D-domain. Bar is 1.5 µm. (B) Statistical quantification for the mean fluorescence intensity (mFI) of Alexa 647-conjugated goat IgG anti human IgM surrogate antigens tethered on the surface of PLB membranes. Each dot represents a single measurement for the mFI of the tethered IgG surrogate antigens by Image J software. Bars represent means ± SD. Two-tailed t tests were performed for statistical comparisons. (C) Shown are representative two-color TIRFM images of BCR (green) or the surrogate antigen (red) microclusters within the contact interface of human Ramos B cell with the PLB membranes tethering the Alexa 647-conjugated goat IgG anti human IgM surrogate antigens. Also shown are the merged images. Bar is 1.5 µm. (D) Statistical quantification for the mFI of BCR microclusters (top panel) or surrogate antigen microclusters (lower panel) within the B cell immunological synapse. Each dot shows one measurement from a single cell. Bars represent means ± SD. Two-tailed t tests were performed for statistical comparisons.

Next we examined the capability of H12-D-domain construct to tether IgG surrogate antigens on Ni^2+^-containing PLB membranes by TIRFM molecule imaging (Fig. 1B). We prepared the Ni^2+^-containing fluid PLB membranes on pre-cleaned cover slides following our published protocol [23]–[26]. We incubated the PLB membranes with 50 nM H12-D-domain construct for 20 min at room temperature (RT). After washing, we incubated the Alexa 647-conjugated goat IgG molecule anti-human IgM as surrogate antigens at 100, 50 or 0 nM on the PLB membranes tethering the H12-D-domain constructs for 20 min at RT as depicted in Figure 1B. After washing, we imaged the distribution of Alexa647-conjugated goat IgG surrogate antigens on PLB membranes by TIRFM. We quantified the amount of these IgG surrogate antigens that were captured on the surface of PLB membranes as mean fluorescence intensity (mFI) using Image J software following our published protocol [24], [26]. We found that these goat IgG surrogate antigens were efficiently captured by the PLB membranes tethering the H12-D-domain construct in a dose dependent manner (Fig. 2A, 2B).

### The Tethered IgG Surrogate Antigens are Biological Active and Induce the Formation and Accumulation of Signaling Active BCR Microclusters

Next we checked if these surrogate antigens of Alexa 647-conjugated goat IgG anti-human IgM molecules that are tethered on the surface of PLB membrane maintained their biological activities to stimulate BCRs. To do that, we used a laboratory human B cell line, Ramos B cell, which expresses human IgM-BCRs. We pre-stained Ramos B cells with Alexa 488-conjugated Fab fragment anti-human IgM Fcμ5 antibody and then placed these B cells on IgG surrogate antigens-containing PLB membranes. After an incubation time of 10 min in a 37°C, 5% CO2 and humidified cell culture incubator, we examined the formation of BCR microcluster, IgG surrogate antigen microclusters, and the accumulation of these BCR and antigen microclusters into the B cell immunological synapse by TIRFM imaging following our published protocol [24], [26]. Not surprisingly, prominent BCR microclusters and IgG surrogate antigen microclusters were observed within the immunological synapse of the B cell contact interface with the IgG surrogate antigen-containing PLB membranes (Fig. 2C). Quantitative analyses following our published protocol suggested that the amount of accumulated BCR and surrogate antigen microclusters within the B cell immunological synapse was also in an obvious dose dependent manner (Fig. 2D). We noticed that antigen microclusters mainly localized in the cSMAC area, while there are always some newly formed BCR microclusters showed up at the pSMAC area of the immunological synapse (Fig. 2C), consistent with the published studies by our studies and those of others [8], [23], [27], [30].

**Figure 3:**
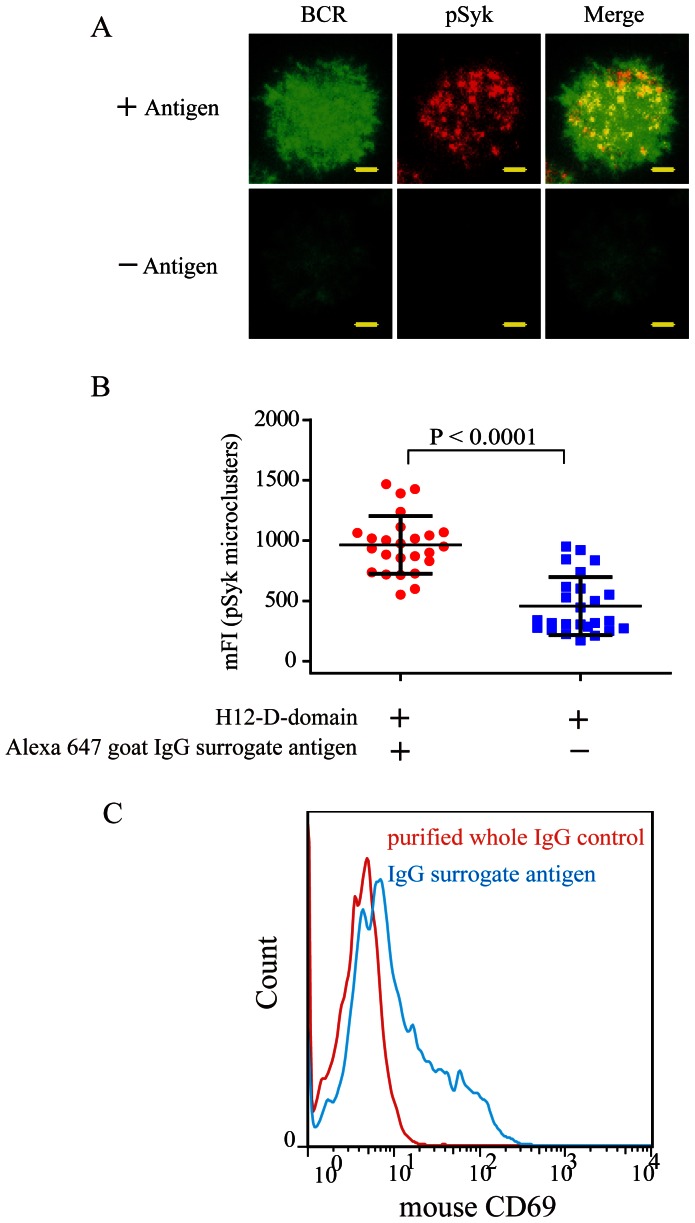
The IgG surrogate antigens tethered on PLB membranes induce the recruitment of pSyk into the B cell immunological synapse and upregulate the surface activation marker CD69 on mice primary B cells. (A) Shown are representative two-color TIRFM images of BCR (green) or pSyk (red) microclusters within the immunological synapse of human Ramos B cells placed on PLB membranes tethering goat IgG anti-human IgM surrogate antigen (top panel) or purified control IgG from goat sera (lower panel) for 10 min. Also shown are the merged images. Bar is 1.5 µm. (B) Statistical quantification for the mFI of pSyk microclusters within the B cell immunological synapse. Each dot shows one measurement from a single cell. Bars represent means ± SD. Two-tailed t tests were performed for statistical comparisons. (C) Flow cytometry analysis for B cell surface activation marker CD69 from mouse primary B cell after a 24-hour incubation on PLB membranes tethering IgG surrogate antigens or the control membranes.

It is believed that the BCR microclusters are the most fundamental platform in the initiation of BCR signaling pathway [40], [41]. The Syk is demonstrated to be an important kinase that is firstly recruited to the immune tyrosine activation motif (ITAM) on the cytoplasmic domain of BCR microclusters [40], [41]. We thus imaged the accumulation of pSyk microclusters within the immunological synapse of human Ramos B cells (Fig. 3). The results show that PLB membranes tethering the IgG surrogate antigens accumulated more pSyk microclusters into the immunological synapse compared to the control PLB membrane containing the H12-D-domain construct alone (Fig. 3A, 3B). Lastly, we evaluated the expression of B cell activation marker CD69 using mouse primary B cells. According to the published studies, B cells increase CD69 expression as an activation marker upon overnight culture with antigens [42]–[44]. We thus incubated mouse primary B cells for 24 hours in cell culture medium with the H12-D-domain-containing PLB membranes tethering goat IgG anti-mouse IgM as surrogate antigens or purified whole IgG from goat sera as a control. We found that mouse primary B cells placed on PLB membrane tethering the IgG surrogate antigens showed the significantly enhanced expression of CD69 activation marker compared to control cells (Fig. 3C). All these observations demonstrated that the IgG surrogate antigens tethered on PLB membranes by H12-D-domain construct are biological active and induce the formation and accumulation of signaling active BCR microclusters within the B cell immunological synapse, and up-regulated the expression of B cell activation marker CD69.

### Robustness of H12-D-domain Based New Method to Tether IgG Surrogate Antigens and IgG Fc Portion-containing Molecules on PLB Membranes

**Figure 4:**
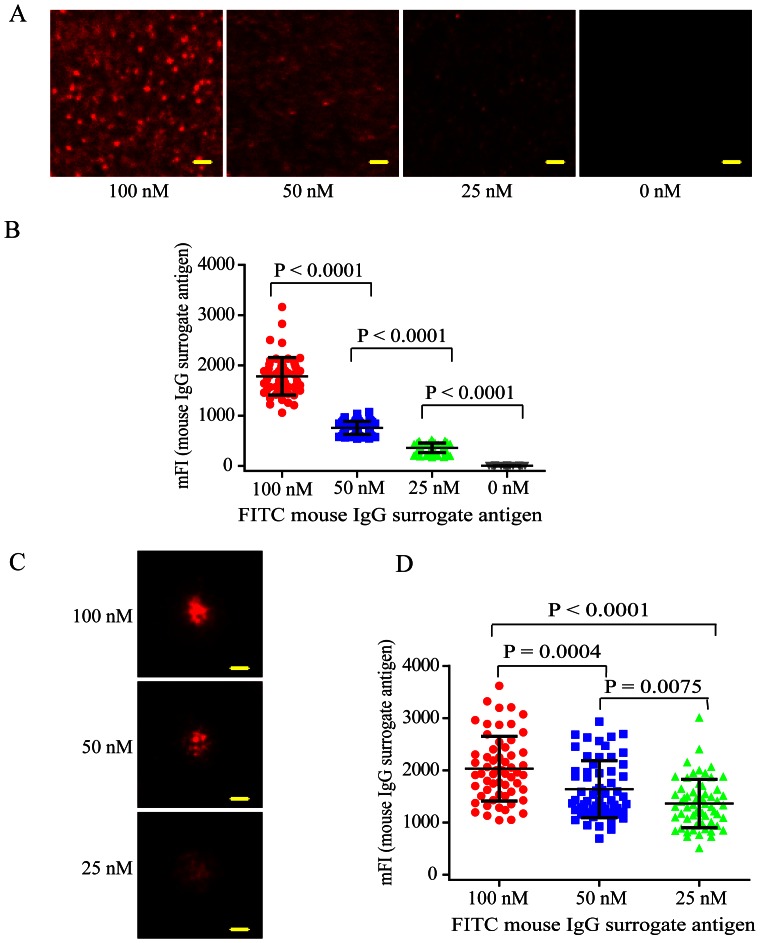
H12-D-domain construct efficiently tethers the IgG surrogate antigens on PLB membranes in a clear-cut dose dependent manner. (A) Shown are representative TIRFM images of FITC-conjugated mouse IgG anti-chicken IgM surrogate antigens tethered on the surface of PLB membranes through H12-D-domain construct. The FITC-conjugated mouse IgG anti-chicken IgM surrogate molecules were pre-incubated at different concentration (100, 50, 25 or 0 nM) with the PLB membranes containing H12-D-domain. Bar is 1.5 µm. (B) Statistical quantification for the mFI of FITC-conjugated mouse IgG anti-chicken IgM surrogate antigens tethered on the surface of PLB membranes. Each dot represents a single measurement for the mFI of the tethered IgG surrogate antigens by Image J software. Bars represent means ± SD. Two-tailed t tests were performed for statistical comparisons. (C) Shown are representative TIRFM images of IgG surrogate antigen microclusters within the contact interface of chicken DT40 B cell with the PLB membranes tethering FITC-conjugated mouse IgG anti-chicken IgM surrogate antigens. Bar is 1.5 µm. (D) Statistical quantification for the mFI of surrogate antigen microclusters within the B cell immunological synapse. Each dot shows one measurement from a single cell. Bars represent means ± SD. Two-tailed t tests were performed for statistical comparisons.

We also tested the robustness of this new method by repeating the above experiments using IgG surrogate antigen molecules and B cells from different species. First, we chose to use the FITC-conjugated mouse IgG anti-chicken IgM as surrogate antigens and a laboratory B cell line of chicken DT40 B cell as responding cells. We incubated the FITC-conjugated mouse IgG surrogate antigens at 100, 50, 25 or 0 nM with the PLB membranes tethering H12-D-domain construct for 20 min at RT. After washing, we placed live chicken DT40 B cells on these IgG surrogate antigen-containing PLB membranes. We incubated them for 10 min in a 37°C, 5% CO2 and humidified cell culture incubator. Similarly we performed the TIRFM imaging and quantified the amount of such IgG surrogate antigens tethered on the surface of PLB membranes (Fig. 4A, 4B). We also examined the accumulation of the surrogate antigen microclusters within the B cell immunological synapse (Fig. 4C, 4D). Consistent with the results of using goat IgG surrogate antigens anti-human IgM and human Ramos B cells, we found that mouse IgG surrogate antigen were captured on the surface of PLB membranes in a clear-cut dose dependent manner (Fig. 4A, 4B). More importantly, prominent surrogate antigen microclusters were formed and accumulated into the B cell immunological synapse also in an obvious dose dependent manner (Fig. 4C, 4D).

We further tested the robustness of the H12-D-domain based new method to tether Alexa647-conjugated donkey IgG anti-mouse IgM, or FITC-conjugated rabbit IgG anti-mouse IgG surrogate antigens on PLB membranes (Fig. S1). As expected, we found that both donkey (Fig. S1A, S1B) and rabbit IgG surrogate antigens (Fig. S1C, S1D) were captured on PLB membranes in a H12-D-domain dependent manner as these IgG surrogate antigens cannot bind to the control PLB membranes without H12-D-domain construct (Fig. S1A–D).

Moreover, we tested the capability of H12-D-domain based new method to tether the recombinant proteins fused with the Fc portion of IgG molecule. The Fc portion of IgG molecule has been widely used as an external domain to help target protein folding and used as an affinity tag in recombinant protein purification. We acquired some purified recombinant IL-1R protein fused with the Fc portion of human IgG molecule (IL-1R-Fc) from Wang and his colleagues [45]. We have these proteins labeled with Alexa 568 fluorophore following our published protocol [23]–[25]. Using Alexa 568-conjugated IL-1R-Fc molecule as an example (Fig. S1E, S1F), we showed that the H12-D-domain construct based new method can efficiently capture the recombinant molecules of interest fused with the Fc portion of IgG molecules, suggesting the broad applications of this new method.

### The H12-D-domain Mediated PLB Membranes Tethering IgG Surrogate Antigens Induced the Formation of Antigen Microclusters within T Cell Immunological Synapse

As mentioned above, the PLB membrane based antigen presentation system has broad applications in B and T lymphocyte cell studies through TIRFM imaging techniques. Having shown the application of the new method for B cells, we further tested the application of this new method on T cells. To do so, we similarly tethered the FITC-conjugated rat IgG anti-mouse CD3 molecular complex surrogate antigens on PLB membranes through H12-D-domain construct, and imaged the formation of antigen microclusters within T cell immunological synapse by TIRFM. Indeed, we found that the H12-D-domain-containing PLB membranes specifically tethered FITC-conjugated rat IgG anti-mouse CD3 surrogate antigens on the surfaces (Fig. S2A, S2B). When encountering these surrogate antigen-containing PLB membranes, laboratory mouse T cell lines, EL 4 T cells, efficiently formed the anti-CD3 surrogate antigen microclusters and accumulated these surrogate antigen molecules into the T cell immunological synapse (Fig. S2C, S2D). These results suggest that IgG surrogate antigens tethered on PLB membranes by H12-D-domain are biologically active and induced the formation and accumulation of surrogate antigen microclusters within the immunological synapse in both B and T lymphocyte cells.

### The H12-D-domain Mediated PLB Membranes only Tether IgG, but not Fab, F(ab’)_2_ or IgM Molecules

**Figure 5:**
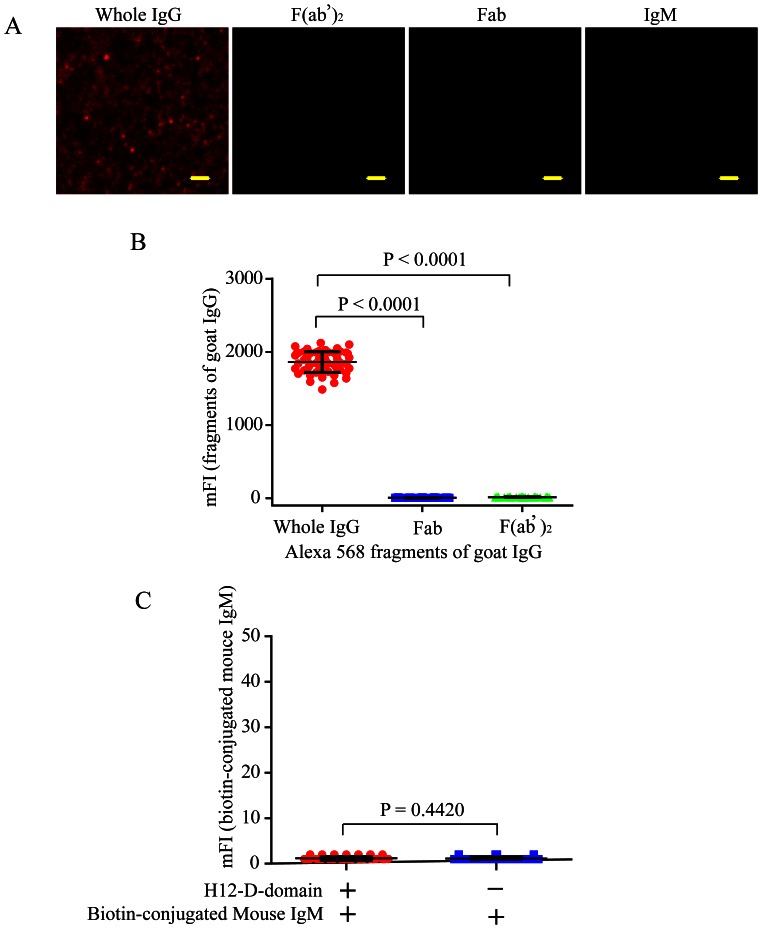
H12-D-domain mediated PLB membranes only tether whole IgG, but not Fab, F(ab’)_2_ or IgM molecules. (A) Shown are representative TIRFM images of Alexa 568-conjugated goat whole IgG, F(ab’)_2_ or Fab fragments of goat IgG molecules, or the biotin-conjugated mouse IgM molecules tethered on the surface of PLB membranes with pre-attached H12-D-domain. The PLB membranes tethering the biotin-conjugated mouse IgM molecules were further incubated with the Alexa 568-conjugated streptavidin for TIRFM imaging. Bar is 1.5 µm. (B) Statistical quantification for the mFI of Alexa 568-conjugated goat whole IgG, F(ab’)_2_ or Fab fragments of goat IgG molecules tethered on the surface of PLB membranes with pre-attached H12-D-domain. (C) Statistical quantification for the mFI of biotin-conjugated mouse IgM molecules tethered on the surface of PLB membranes with or without pre-attached H12-D-domain. The PLB membranes tethering the biotin-conjugated mouse IgM molecules were further incubated with the Alexa 568-conjugated streptavidin for TIRFM imaging. In both B and C, each dot represents a single measurement for the mFI of the tethered IgG surrogate antigens by Image J software. Bars represent means±SD. Two-tailed t tests were performed for statistical comparisons.

We are cautious on the application of H12-D-domain mediated PLB membrane antigen presentation system on the lymphocyte studies, especially in the case of B cell studies because there are usually surface expressed IgM or IgG on B cell. Thus, we further compared the capability of H12-D-domain construct mediated PLB membranes tethering different fragments of IgG molecules including goat whole IgG, Fab or F(ab’)_2_ fragments of goat IgG molecules. The results suggested that only whole IgG, but not Fab, nor F(ab’)_2_ fragment of IgG molecules were efficiently tethered on the PLB membranes through the H12-D-domain construct (Fig. 5A, 5B), suggesting that the H12-D-domain construct cannot interact with the Fab or the F(ab’)_2_ fragment of the surface expressed IgM or IgG molecules on B cells. Similarly, we found that only IgG molecules but not IgM molecules were efficiently tethered on the PLB membranes (Fig. 5C). Since we do not expect the T cells or other type of cells will encounter more complicated problems than the B cells as only B cells express surface immunoglobulins, we anticipate that the application of our new method are broad.

### IgG Surrogate Antigens Tethered by H12-D-domain Show Better Lateral Mobility and are More Uniform on PLB Membranes than the Ones Tethered by Streptavidin

**Figure 6:**
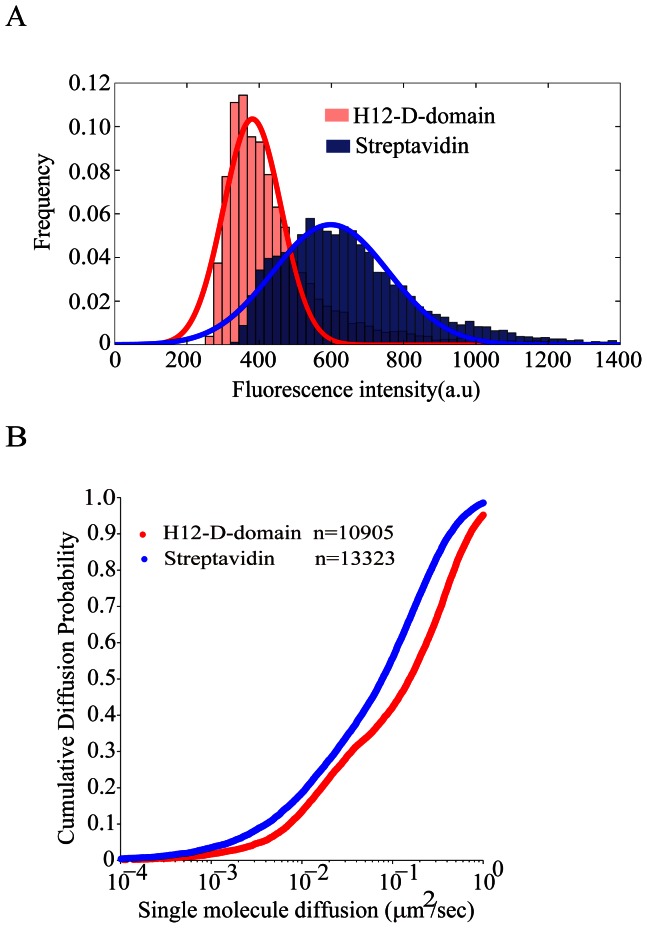
The IgG surrogate antigens tethered by H12-D-domain show more uniform distribution with better lateral mobility than the ones tethered by streptavidin. (A) Shown is the distribution of ﬂuorescence intensities (FI) of 10905 IgG surrogate antigen molecules tethered by H12-D-domain (red color) versus 13323 IgG surrogate antigen molecule tethered by streptavidin (blue color) on PLB membranes with a Gaussian fit (red or blue colored curve respectively) to the histogram plot. (B) Cumulative diffusion probability plot of all the calculated instant diffusion coefficients of 10905 IgG surrogate antigen molecules tethered by H12-D-domain (red color) versus 13323 IgG surrogate antigen molecule tethered by streptavidin (blue color) on PLB membranes.

A unique feature of molecules tethered on PLB membranes are that all these molecules show mobile Brownian diffusion feature, mimicking the situation of the antigens tethered on the plasma membrane of antigen presenting cells. In our new method, the IgG arrogate molecule is bound to the H12-D-domain construct on PLB membrane in an exact 1 to 1 ratio, thus we assume that the IgG molecules tethered by H12-D-domain will show more uniform distribution and better mobility feature than the IgG molecules tethered by streptavidin, which can bind up to four molecules of biotinylated IgGs. To test that, we conjugated both biotin and Alexa 568 to goat whole IgG anti-human IgM antibodies and have these antibodies tethered on either Ni^2+^-containing PLB membranes by H12-D-domain or biotin-containing PLB membranes by streptavidin. Then, we performed the single molecule tracking experiments using TIRFM imaging experiment at RT, and analyzed these TIRFM images by a Matlab driven algorithm for fluorescence intensity (FI) and instant diffusion coefficient of each IgG molecules following our published protocol [23]–[25]. Indeed, we found that in both cases the biotin and Alexa 568 conjugated IgG molecules tethered on PLB membranes appeared as obvious individual mobile spots of similar FI as shown in the FI histogram (Fig. 6A), consistent with a typical FI histogram of membrane-bound antigens in the published studies. Importantly, it is clear that the distribution of IgG surrogate antigens tethered by H12-D-domain is more uniform with a much narrower full width at half maximal of the Gaussian function fitted FI histogram plot than the case of the IgG surrogate antigens tethered by streptavidin (Fig. 6A). Moreover, we noticed that there is an obvious shift to the end with high FI value of the FI histogram plot produced from the streptavidin-bearing PLB membranes compared to the case of the H12-D-domain-bearing PLB membranes (Fig. 6A), suggesting the formation of oligomerized IgG molecules on streptavidin-bearing PLB membranes. These FI based single IgG molecule analyses support our speculation that the 1 to 1 binding stoichiometry of H12-D-domain to IgG facilitates a more uniform distribution of IgG surrogate antigens on PLB membranes (Fig. 6A). We further calculated the instant diffusion coefficients of at least ten thousand of individual IgG molecules through the analyses of the short-range mean-square displacement (MSD) of each individual IgG molecule trajectories (Fig. 6B). The results suggested that indeed the IgG surrogate antigens tethered by H12-D-domain exhibit better lateral mobility than the IgG molecules tethered by streptavidin (Fig. 6B), which further supported our speculation.

### IgG Surrogate Antigens Tethered by H12-D-domain Enhance the Accumulation of BCR and pSyk into Immunological Synapse than the Ones Tethered by Streptavidin

**Figure 7:**
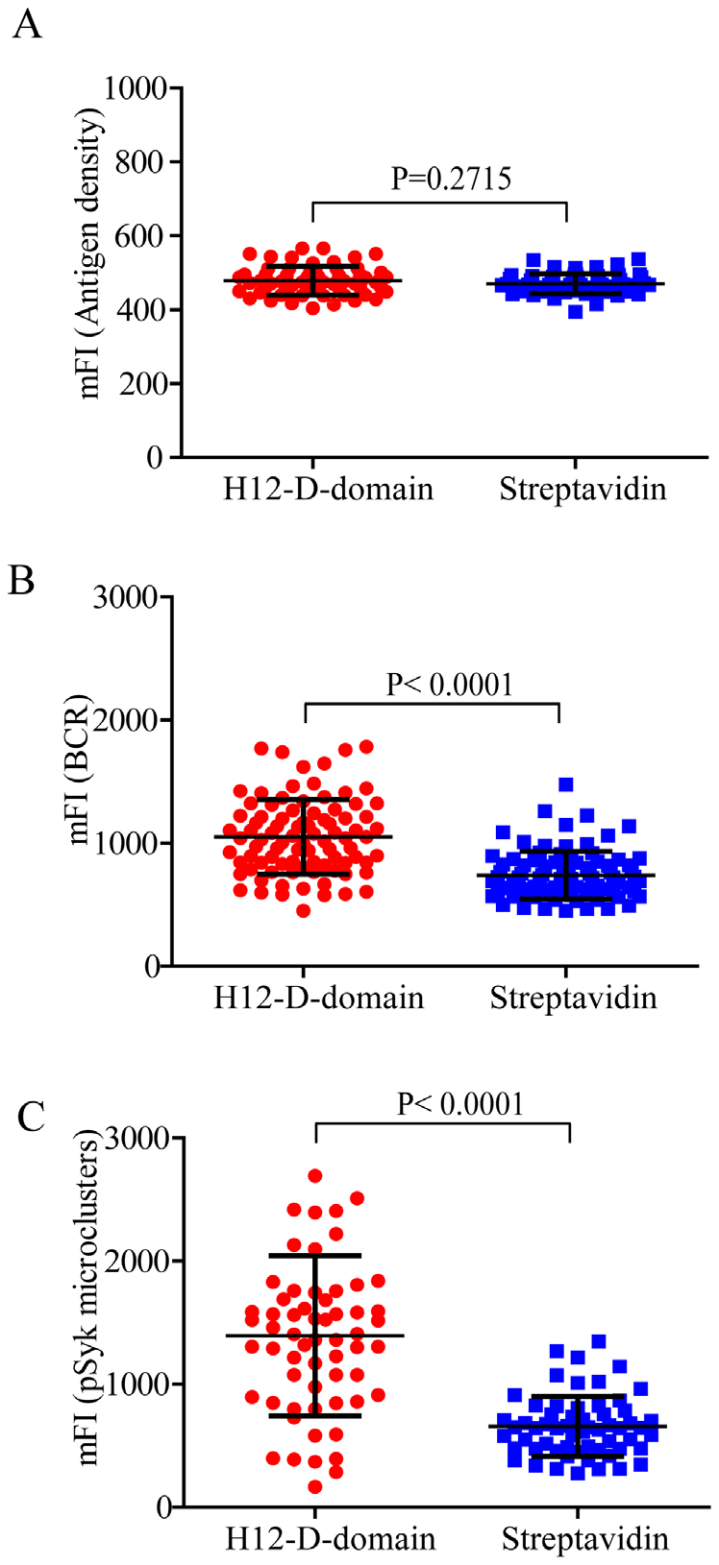
IgG surrogate antigens tethered by H12-D-domain enhance the accumulation of BCR and pSyk into B cell immunological synapse than the ones tethered by streptavidin. (A) Statistical quantification for the mean fluorescence intensity (mFI) of biotin and Alexa 568-conjugated goat IgG anti human IgM surrogate antigens tethered on the surface of PLB membranes by either H12-D-domain (red color) or streptavidin (blue color). Each dot represents a single measurement for the mFI of the tethered IgG surrogate antigens by Image J software. Bars represent means ± SD. Two-tailed t tests were performed for statistical comparisons. (B) Statistical quantification for the accumulation of human IgM-BCRs into the immunological synapse as measured by the mFI of BCR within the immunological synapse from Ramos human B cells that were placed on PLB membranes presenting the same amount of IgG surrogate antigen as shown in A that were tethered by either H12-D-domain or streptavidin. Each dot represents a single measurement for the mFI of IgM-BCRs by Image J software. Bars represent means ± SD. Two-tailed t tests were performed for statistical comparisons. (C) Statistical quantification for the accumulation of pSyk signaling molecules into the immunological synapse as measured by the mFI of BCR within the immunological synapse from Ramos human B cells that were placed on PLB membranes presenting the same amount of IgG surrogate antigen as shown in A that were tethered by either H12-D-domain or streptavidin. Each dot represents a single measurement for the mFI of pSyk by Image J software. Bars represent means ± SD. Two-tailed t tests were performed for statistical comparisons.

Next we tested the potency of recruiting BCRs and pSyk molecules into B cell immunological synapse by the same batch of IgG surrogate antigens that are tethered on the PLB membranes by either H12-D-domain or streptavidin. Since it is well documented the antigen concentration on the PLB membranes significantly affects the formation of B cell immunological synapse [23]–[25], we first did a series of titration experiments for the amount of biotin and Alexa 568 conjugated goat whole IgG anti-human IgM surrogate antigens on both H12-D-domain-bearing and streptavidin-bearing PLB membranes. We used the condition that presents comparable amount of IgG molecules on these two types of PLB membranes as quantified by the mFI of the biotin and Alexa 568 conjugated goat whole IgG anti-human IgM surrogate antigens (Fig. 7A). We have the Ramos human B cells pre-stained with Alexa 488-conjugated Fab fragment anti-human IgM Fcμ5 antibody and then placed on these two types of PLB membranes. After an incubation time of 10 min in a 37°C, 5% CO2 and humidified cell culture incubator, we examined the accumulation of human IgM-BCRs and pSyk molecules within the immunological synapse by TIRFM imaging. Indeed, the quantitative analyses suggested that IgG surrogate antigens that are tethered by H12-D-domain enhance the accumulation of BCR and pSyk into B cell immunological synapse than IgG molecules tethered by streptavidin (Fig. 7B, 7C).

## Discussion

In this report, we introduced a new and robust method of tethering IgG surrogate antigens on the surface of planar lipid bilayer (PLB) membranes. The significantly increased needs of TIRFM based live cell and single molecule imaging applications require the development of new and robust method, as an alternative choice, to tether IgG surrogate antigens or molecules containing IgG Fc portion (Fc fusion proteins, like ICAM-1-Fc) on PLB membranes. Currently, there are generally two methods to tether molecules of interest on PLB membranes through specific affinity tags. The first method is to use Ni^2+^-containing PLB membranes. In this method, the polyhistidine tagged molecules of interest can be easily tethered. For example, in our earlier studies, we have been able to tether the polyhistidine tagged hapten molecule 4-hydroxy-3-iodo-5-nitrophenyl (NIP) on Ni^2+^-containing PLB membranes to address the activation of B lymphocyte cells [24]. However, it would be difficult to directly tether IgG molecules on this type of PLB membranes without providing IgG molecules a polyhistidine tag.

The second method is to use the biotin-containing PLB membranes. In this case, streptavidin molecules are commonly used as a mediator to tether biotin-conjugated IgG molecules on PLB membranes. Although this method has been widely used by others and us [8], [18]–[26], there are two substantial problems: (1) Biotin-conjugated IgG molecules instead of intact IgG are used in this method. When we biotinylating the IgG molecules, the biotin molecules can be covalently conjugated to either the variable region or the constant region of IgG molecules. As a result, the biotinylated IgG molecules can be tethered on biotin-containing PLB membranes in an “upside down” manner which will likely make these IgG molecules inaccessible and non-functional; (2) When using streptavidin to tether biotinylated IgG molecules on biotin-containing PLB membranes, multiple IgG molecules can bind to one individual streptavidin molecule due to the fact that one streptavidin can bind up to four biotin molecules with very high affinity. In this case, the biotinylated IgG molecules can be tethered on biotin-containing PLB membranes in a “multi-molecule” manner, like two or more IgG molecules bind to one streptavidin, which makes the IgG surrogate antigens non-uniform and complicated.

In our new method, we combined these two different approaches and provide an alternative choice for the tethering of intact IgG surrogate antigens on Ni^2+^-containing PLB membranes. To identify an appropriate bridge molecule to tether the intact IgG surrogate antigens on PLB membranes, we targeted to the well-studied IgG receptor from microbial proteins that are expressed by certain types of the gram-positive bacteria. Such proteins are known for their capability to bind to the constant fragment (Fc portion) of IgG molecules with high affinity and specificity, like Protein G from group G and C strains of streptococci [34], [35], Protein M from *Streptococcus pyogenes* [37], and Protein A from *Staphylococcus aureus* [36].

We focused on Protein A as it has been extensively studied. It is known that one protein A molecule has four or five 56 to 61 aa residue Ig binding domains (domain A, B, C, D and E) [28]. Since the binding mechanism of the D domain of staphylococcal protein A (SpA) to the Fc portion of IgG molecule or the Fab V_H_-3 region of both IgG and IgM molecules was well studied [38], we exclusively explored the application of SpA D domain to our new method in this report. However we did not exclude the possibility that other IgG binding proteins, like protein G or protein M, can be similarly modified and used in PLB membrane antigen presentation systems.

The SpA D-domain adopts three alpha-helix bundles [46]. Early studies well demonstrated at atomic level the structural interactions of SpA Ig binding domain with IgG constant region [47] or Fab V_H_-3 region of both IgG and IgM molecules [39]. It is documented that amino acid residues round alpha-helix H1 mediates the binding of SpA D-domain to IgG constant region, and among these residues, the Glutamine (Q) 9 and 10 are the most critical ones; in contrast, the amino acid residues round alpha-helix H2 and H3 mediate the binding of SpA D-domain to V_H_-3 region of both IgG and IgM molecules, and among them, the Aspartate (D) 36 and 37 between the linker region of H2 and H3 helical bundle are the most critical residues [38]. In this report, we have been trying to seek a SpA D-domain mutant maintaining the binding capability to IgG constant region (Fc portion), but eliminating the binding capability to the Fab V_H_-3 region of both IgG and IgM molecules. Based on the elegant early studies as mentioned above, we choose to mutate the DD (aa36–37) to AA (aa36–37) in the original D domain sequences. According to the published studies from others [38], [39] and our own data in this report, this mutant of SpA D-domain eliminated its binding capability to Fab V_H_-3 region of IgG and IgM molecules. In our proposed method, such modification to SpA D-domain is absolutely needed as mutating the DD (aa36–37) to AA (aa36–37) in SpA D-domain can efficiently eliminate the super-antigen ability of SpA D-domain to stimulate the B cell receptors (BCRs) hyper-activation and the subsequent B cell proliferation [48]. We also fused the mutant SpA D-domain a 12 aa polyhistidine tag at the N-terminus to provide its capability to specifically tether to Ni^2+^-containing PLB membranes.

We extensively examined the specificity and capability of H12-D-domain construct to tether IgG molecules on Ni^2+^-containing PLB membranes. H12-D-domain construct showed potent abilities to tether whole IgG surrogate antigens on PLB membranes. In contrast, Fab and F(ab’)_2_ fragment of IgG molecules, or IgM molecules cannot be tethered on the PLB membranes by H12-D-domains. It is known that protein A can bind IgG Fc portion with a very high equilibrium association constant (K_A_) value of 2.8∼2.9×10^8^ M^−1^ and a very low equilibrium dissociation constant (K_D_) value of 3.4∼3.6 nM [49]–[51]. Our synthesized H12-D-domain has only one IgG Fc portion binding domain, D-domain, from protein A. It is reported that the K_A_ value of a single IgG binding domain to IgG molecule is 5.9∼10×10^7^ M^−1^ with a K_D_ value of 10∼16.9 nM [50]. Although the affinity of H12-D-domain to IgG is lower than the case of protein A to IgG, it is not likely that the tethered IgG surrogate molecule on PLB membranes can dissociate with H12-D-domain and interaction with the low affinity Fc receptor, FcγRIIB, which are expressed on the surface of many types of immune cells. Our speculation is based on the following two facts: (1) In our experimental system, we generally used the H12-D-domain at 50 nM and the IgG surrogate antigen at 50 or 100 nM, which is a concentration far above the K_D_ value of IgG and H12-D-domain (10∼16.9 nM) [50]; (2) The affinity constant of IgG molecules to FcγRIIB is extremely low (K_A_, 3.97∼5.99×10^5^ M^-1^) and K_D_ is extremely high at 1670 ∼ 2520 nM [52].

Our synthesized H12-D-domain has one IgG Fc portion binding region per early structural studies [47]. Thus H12-D-domain bind to IgG surrogate antigen in a 1 to 1 ratio, which makes the binding of IgG and H12-D-domain-bearing PLB membranes simple and specific, rendering uniformly distributed IgG molecules on PLB membranes with good lateral mobility. Another unique feature of our presented new method is that IgG molecules bind to H12-D-domain with their Fc portion. We thus speculate that IgG surrogate antigens tethered on PLB membranes show their orientation as “stand-up” status, which will likely facilitate their capability to recognize and stimulate the antigen receptors on the opposing membrane of a cell. Such speculation is supported by the experiments examining the activation of lymphocyte cells encountering these IgG surrogate antigens tethered on PLB membranes using our new method versus the conventional method involving streptavidin as a bridge molecule.

We used B and T lymphocyte cells and TIRFM based live cell imaging to examine the efficacy of these tethered IgG molecules as surrogate antigens. We found that these IgG surrogate antigens strongly activated both B and T cells by inducing the formation and accumulation of signaling active antigen receptor microclusters, IgG surrogate antigen microclusters and pSyk signaling molecule microclusters within the immunological synapse. All these observations are consistent with the early phase activation events of lymphocyte cells encountering the antigens tethered on PLB membranes using conventional methods [9], [40]. More importantly, mouse primary B cells efficiently up-regulated the surface activation marker CD69 upon an overnight culture with these IgG surrogate antigens. All these data suggested that the mutant H12-D-domain derived from *Staphylococcus aureus* is a good bridge molecule to tether intact IgG surrogate antigens on the surface of PLB membranes and meanwhile well maintain the biological activity of these IgG surrogate antigens.

This new method of using H12-D-domain to tether IgG surrogate antigens on PLB membranes can be applied to IgG molecules from a diverse range of species. We found that IgG molecules from mouse, rat, rabbit, goat and donkey can be efficiently tethered to PLB membranes through H12-D-domain. More importantly, many recombinant molecules fused with the Fc portion of IgG molecules, like recombinant ICAM-1-Fc molecule, are currently widely used in cell biology studies. We speculate that using this new method these IgG Fc portion fused molecules can be tethered on PLB membranes. Indeed, we showed that recombinant IL-1 receptor protein fused with the Fc portion of human IgG molecule can be efficiently tethered on the surface of PLB membranes through H12-D-domain molecules, suggesting the much broader application of this new method. In this short report, we just tested the responses of B and T lymphocyte cells encountering IgG surrogate antigens tethered on PLB membranes. We guess that antibody and antigen immune complexes tethered on the PLB membrane using our new method can be used as good stimuli to activate the IgG Fc portion receptors on immune cells including macrophages, neutrophils and dendritic cells.

We do not expect that our new method can fit to all the related applications. We listed below the potential caveats and boundaries of this new method. (1) It’s reported that the D-domain of *Staphylococcus aureus* can also bind to von Willebrand factor (vWF) molecule on cell surfaces [53], [54]. vWF is a large multifunctional glycoprotein characterized by high molecular weight up to 15 million Daltons. The expression of vWF is restricted to endothelial cells and megakaryocyte cells. Endothelial vWF is secreted constitutively in the blood or in the sub-endothelial matrix [53], [54]. Thus, we discourage the investigators using this method to vWF related studies in endothelial cells and megakaryocyte. (2) Due to the fact that H12-D-domain can bind to the Fc region of IgG, we don’t recommend the H12-D-domain based PLB membrane to be used for studies involving memory B cells expressing IgG-BCRs. (3) We always suggest to include a control group of PLB membranes presenting the H12-D-domain plus the isotype matched control IgG molecules to monitor the response and behavior of the cells in case there are some unexpected cell surface receptors showing the interaction with the H12-D-domains. In our new method, we always block the H12-D-domain molecules that are not bound to the IgG surrogate antigens with excessive amount of non-targeting control IgG molecules in the experiments involving intracellular staining steps. Since the H12-D-domain construct that we used in this new method is monovalent, we do not expect that the H12-D-domain can get aggregated when encountering excessive amount of IgG molecules. Such blocking step seems to work well. For example, in our experiment, we blocked the H12-D-domain molecules on the PLB membranes tethering or not tethering goat anti-mouse IgG surrogate antigens with the purified IgG molecules from goat sera. We examined the response of mouse A20 B cells expressing IgG-BCRs on these two types of PLB membranes. To our surprise, we found that the PLB membranes presenting the H12-D-domain plus the control IgG molecules cannot activate the A20 B cells (data not shown), suggesting that the H12-D-domain constructs on PLB membranes upon blocking with non-targeting control IgG may lose the ability to bind to the IgG-BCRs on A20 B cells.

Overall, we provide a new and robust method to tether IgG surrogate antigens or other recombinant protein molecules fused with IgG Fc portion on the surface of Ni^2+^-containing PLB membranes. This innovative method will be helpful to boost the studies on the complicated receptor and ligand interactions during cell-cell crosstalk by TIRFM based imaging experiments.

## Supporting Information

Figure S1 The new method based on H12-D-domain construct robustly tethers IgG surrogate molecules from different species and other recombinant molecules containing the IgG Fc portion to PLB membranes. (A), (C) and (E) Shown are representative TIRFM images of Alexa647-conjugated donkey IgG anti-mouse IgM surrogate antigens (A), FITC-conjugated rabbit IgG anti-mouse IgM surrogate antigens (C), or Alexa 568-conjugated IL-1R-Fc molecule (E) tethered on the surface of PLB membranes with (left panel) or without (right panel) H12-D-domain molecules. Bar is 1.5 µm. (B), (D) and (F) Statistical quantification for the mFI of Alexa647-conjugated donkey IgG anti-mouse IgM surrogate antigens (B), FITC-conjugated rabbit IgG anti-mouse IgM surrogate antigens (D), or Alexa 568-conjugated IL-1R-Fc molecule (F) tethered on the surface of PLB membranes with (red closed circle) or without (blue closed square) H12-D-domain molecules.
(TIF)

Figure S2 IgG surrogate antigens tethered on PLB membranes induce the formation of antigen microclusters within T cell immunological synapse. (A) Shown are representative TIRFM images of FITC-conjugated rat IgG anti-mouse CD3 molecular complex surrogate antigens tethered on the surface of PLB membranes with (top panel) or without (lower panel) the pre-attached H12-D-domain construct. Bar is 1.5 µm. (B) Statistical quantification for the mFI of FITC-conjugated rat IgG anti-mouse CD3 molecular complex surrogate antigens tethered on the surface of PLB membranes with or without the pre-attached H12-D-domain construct. Each dot represents a single measurement for the mFI of the tethered IgG surrogate antigens by Image J software. Bars represent means ± SD. Two-tailed t tests were performed for statistical comparisons. (C) Shown are representative TIRFM images of IgG surrogate antigen microclusters within the contact interface of mouse EL4 T cells with the PLB membranes tethering FITC-conjugated mouse IgG anti-chicken IgM surrogate antigens with H12-D-domain (top panel) or without (lower panel) the linker H12-D-domain construct. Bar is 1.5 µm. (D) Statistical quantification for the mFI of surrogate antigen microclusters within the T cell immunological synapse. Each dot shows one measurement from a single cell. Bars represent means ± SD. Two-tailed t tests were performed for statistical comparisons.
(TIF)
