# Supplementary figures and images for: A New and Robust Method of Tethering IgG Surrogate Antigens on Lipid Bilayer Membranes to Facilitate the TIRFM Based Live Cell and Single Molecule Imaging Experiments
Source: PLoS One. 2013 May 22;8(5):e63735. doi: 10.1371/journal.pone.0063735 (PMC3661735; doi:10.1371/journal.pone.0063735)

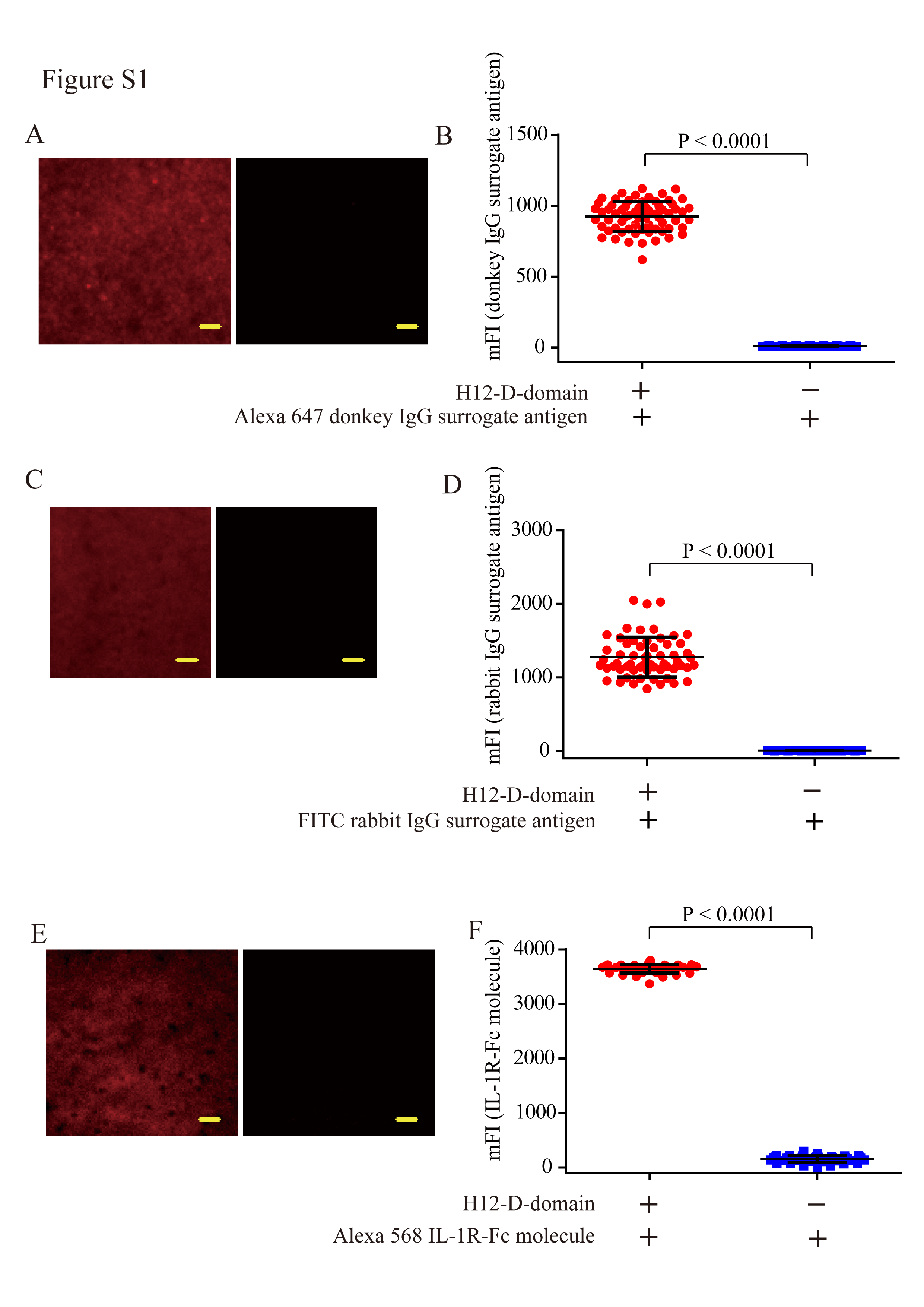

Supplement: Figure S1 — The new method based on H12-D-domain construct robustly tethers IgG surrogate molecules from different species and other recombinant molecules containing the IgG Fc portion to PLB membranes. (A), (C) and (E) Shown are representative TIRFM images of Alexa647-conjugated donkey IgG anti-mouse IgM surrogate antigens (A), FITC-conjugated rabbit IgG anti-mouse IgM surrogate antigens (C), or Alexa 568-conjugated IL-1R-Fc molecule (E) tethered on the surface of PLB membranes with (left panel) or without (right panel) H12-D-domain molecules. Bar is 1.5 µm. (B), (D) and (F) Statistical quantification for the mFI of Alexa647-conjugated donkey IgG anti-mouse IgM surrogate antigens (B), FITC-conjugated rabbit IgG anti-mouse IgM surrogate antigens (D), or Alexa 568-conjugated IL-1R-Fc molecule (F) tethered on the surface of PLB membranes with (red closed circle) or without (blue closed square) H12-D-domain molecules. (TIF) [file pone.0063735.s001.tif]

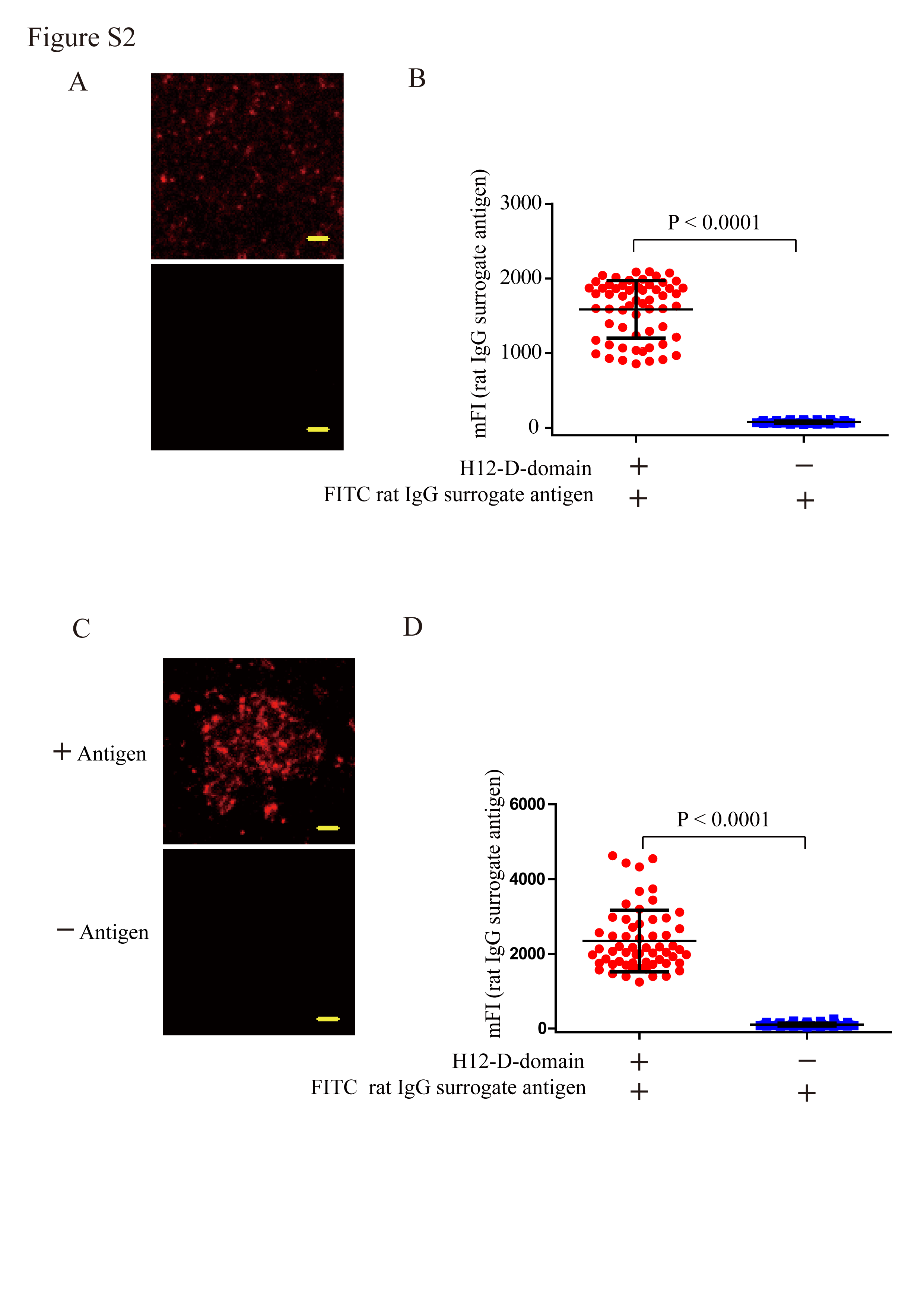

Supplement: Figure S2 — IgG surrogate antigens tethered on PLB membranes induce the formation of antigen microclusters within T cell immunological synapse. (A) Shown are representative TIRFM images of FITC-conjugated rat IgG anti-mouse CD3 molecular complex surrogate antigens tethered on the surface of PLB membranes with (top panel) or without (lower panel) the pre-attached H12-D-domain construct. Bar is 1.5 µm. (B) Statistical quantification for the mFI of FITC-conjugated rat IgG anti-mouse CD3 molecular complex surrogate antigens tethered on the surface of PLB membranes with or without the pre-attached H12-D-domain construct. Each dot represents a single measurement for the mFI of the tethered IgG surrogate antigens by Image J software. Bars represent means ± SD. Two-tailed t tests were performed for statistical comparisons. (C) Shown are representative TIRFM images of IgG surrogate antigen microclusters within the contact interface of mouse EL4 T cells with the PLB membranes tethering FITC-conjugated mouse IgG anti-chicken IgM surrogate antigens with H12-D-domain (top panel) or without (lower panel) the linker H12-D-domain construct. Bar is 1.5 µm. (D) Statistical quantification for the mFI of surrogate antigen microclusters within the T cell immunological synapse. Each dot shows one measurement from a single cell. Bars represent means ± SD. Two-tailed t tests were performed for statistical comparisons. (TIF) [file pone.0063735.s002.tif]
